# Brain Antigens Stimulate Proliferation of T Lymphocytes With a Pathogenic Phenotype in Multiple Sclerosis Patients

**DOI:** 10.3389/fimmu.2022.835763

**Published:** 2022-01-31

**Authors:** Assaf Gottlieb, Hoai Phuong T. Pham, John William Lindsey

**Affiliations:** ^1^ Center for Precision Health, School of Biomedical Informatics, University of Texas Health Science Center at Houston, Houston, TX, United States; ^2^ Division of Multiple Sclerosis and Neuroimmunology, Department of Neurology, McGovern Medical School, University of Texas Health Science Center at Houston, Houston, TX, United States

**Keywords:** multiple sclerosis, autoimmunity, autoantigen, T lymphocytes, T-cell receptor

## Abstract

A method to stimulate T lymphocytes with a broad range of brain antigens would facilitate identification of the autoantigens for multiple sclerosis and enable definition of the pathogenic mechanisms important for multiple sclerosis. In a previous work, we found that the obvious approach of culturing leukocytes with homogenized brain tissue does not work because the brain homogenate suppresses antigen-specific lymphocyte proliferation. We now report a method that substantially reduces the suppressive activity. We used this non-suppressive brain homogenate to stimulate leukocytes from multiple sclerosis patients and controls. We also stimulated with common viruses for comparison. We measured proliferation, selected the responding CD3+ cells with flow cytometry, and sequenced their transcriptomes for mRNA and T-cell receptor sequences. The mRNA expression suggested that the brain-responding cells from MS patients are potentially pathogenic. The T-cell receptor repertoire of the brain-responding cells was clonal with minimal overlap with virus antigens.

## 1 Introduction

Multiple sclerosis (MS) is a human disease characterized by inflammation and demyelination in the central nervous system (CNS), which often causes significant neurologic deficits. MS is thought to be an autoimmune disease and is usually treated with agents that affect some aspect of immune function. Unlike some other autoimmune diseases, the target autoantigen in MS is unknown. There are no diagnostic autoantibodies, and a target T-cell antigen has not been identified. The major structural proteins of myelin, such as myelin basic protein (MBP), proteolipid protein (PLP), or myelin oligodendrocyte glycoprotein (MOG), are obvious candidate autoantigens. These proteins have been extensively investigated (1–5), but we doubt that they are the primary autoantigen. Recent elegant studies have suggested two non-myelin target antigens (6, 7).

In addition to not knowing the target antigen, our understanding of the etiology, pathogenesis, and disease mechanism in MS is incomplete and evolving. Much of our current understanding comes from animal models or from studies of human peripheral blood mononuclear cells (PBMC) stimulated with MBP, PLP, or MOG. These suggest that MS is a T-cell-mediated disease, with the disease-causing T cells secreting the cytokines interleukin-17 (IL17), interferon-γ (IFNγ), and granulocyte-macrophage colony stimulating factor (GM-CSF) (8–11). These cells are often defined by surface expression of the chemokine receptor CCR6 (8, 12).

Focused investigations of the cells present in the cerebrospinal fluid (CSF) of MS patients support the importance of these three cytokines (12, 13), but recent, unbiased examination of gene expression in cerebrospinal fluid cells with RNA-Seq has expanded our concept of the pathogenic phenotype of MS T cells and identified additional transcripts upregulated in MS, such as IL32 (14–16). These RNA-Seq studies also suggest that the pathogenic cells in MS may be T follicular helper (Tfh) cells (14, 17).

A method to efficiently test T-cell responses to a broad range of brain antigens would be useful both for defining the T-cell autoantigen targets in MS and the pathogenic phenotype of the autoreactive cells. The antigen specificity of B cells can be tested against a wide range of antigens, using immunohistochemistry, Western blots, expression libraries, or antigen microarrays (18–21). However, the methods suitable for detecting autoantibodies are difficult to apply to T cells. In fact, in previous work, we found that the simple approach of culturing PBMC with whole brain homogenate suppressed rather than stimulated lymphocyte proliferation (22). In this work, we report methods to reduce the suppressive activity and measure proliferation of PBMC to an extract of brain homogenate. We report the proliferative response, the mRNA expression, and the T-cell receptor (TCR) repertoire of the responding T cells.

## 2 Materials and Methods

### 2.1 PBMC

Blood from 5 MS patients was obtained by phlebotomy, with 40 to 80 ml collected into heparinized tubes. Four patients were on treatment with ocrelizumab, and one patient was newly diagnosed and untreated. Three patients were female, and two were male. The mean age was 29 years, with a range of 18 to 38. PBMC were isolated by centrifugation over a Lymphoprep density gradient in SepMate tubes (StemCell Technologies, Cambridge, MA), washed in phosphate buffered saline, and resuspended in media. PBMC from controls were obtained from the Gulf Coast Regional Blood Center as buffy coats from donated blood and from healthy subjects recruited in the medical center. PBMC were used fresh on the day of isolation.

### 2.2 Antigens

Human brain specimens were obtained from autopsy tissue from three different individuals deceased of non-neurologic disease. No demographic details on the brain donors are available. Brain tissue from cerebral cortex including both white and gray matter was homogenized in a Dounce homogenizer in 10 mM Tris at a concentration of 0.125 g of wet tissue per milliliter, with a typical weight of 2 to 10 g of brain tissue per preparation. The homogenate was centrifuged at 16,000 *g* for 5 min, and the supernatant and pellet were separated and the pellet was resuspended at the original volume in PBS. The supernatant (supe) had neither stimulatory nor suppressive activity, and the resuspended pellet (BH) suppressed PBMC proliferation *in vitro* as previously observed (22). To remove the suppressive activity, the pellet was treated with DNase for 30 min, exposed to 40 mM NaOH, and then neutralized. The alkali exposure removes 70% to 90% of the suppressive activity. The homogenate was then extracted three times with ether:ethanol in a 3:2 ratio, resuspended twice in PBS, and dialyzed against PBS to remove the organic solvents. The DNase treatment is essential to maintain the pellet in a state where it can be resuspended after each centrifugation. After dialysis, we adjusted the volume to the original starting volume, homogenized again, and measured the protein concentration with the bicinchoninic acid assay. The denatured, organic-extracted homogenate is termed Bd (**Supplementary Figure 1A**). On visualization with Coomassie-stained polyacrylamide gels, the protein bands in Bd and BH are similar (**Supplementary Figure 1B**).

Epstein–Barr virus (EBV) was isolated from the B95.8 cell line as previously described (23). Autologous, EBV-infected lymphoblastoid cell lines (LCL) were generated in our laboratory (24). Varicella Zoster virus (VZV) was a live attenuated virus formulated for vaccine (Zostavax, Merck and Co., Whitehouse Station, NJ). Influenza A 2 mg/ml was purchased from Charles River Laboratories (Wilmington, MA). *Candida albicans* antigen was purchased from Greer Labs (Lenoir, NC, catalog number M15A05).

### 2.3 Cell Culture

The majority of experiments were done with PBMC suspended at a concentration of 10^6^ cells/ml in AIM-V media (Gibco, Grand Island, NY) supplemented with 10% autologous human plasma. Autologous plasma was obtained from the same blood sample as the PBMC. Some initial experiments also used RPMI-1640 media (Sigma, St. Louis, MO) supplemented with 10% fetal bovine serum (FBS, Gibco). Antigen concentrations were determined empirically by testing several concentrations of each antigen with multiple subjects. Brain antigens were used at 80 µg protein/ml. This concentration produced maximal stimulation and did not interfere with subsequent flow cytometry. EBV was used at 3.5 µg protein/ml. VZV and influenza were used at 0.5 µl/ml, and candida was used at 5 µl/ml. Cells were cultured in triplicate in 96-well round bottom plates with 0.2 ml/well for proliferation assay, and in bulk with up to 10 ml/well in 6-well plates for flow cytometry. Cells for flow cytometry were labeled with carboxyfluorescein succinimidyl ester (CFSE) before antigen stimulation. This dye crossed the cell membrane and covalently couples to intracellular amines. The concentration of CFSE in the cells decreases with each cell division. The number of cells stimulated with each antigen ranged from 5 × 10^6^ to 2 × 10^7^. For the proliferation assay, tritiated thymidine was added after 4 days with cells harvested and counted at 5 days. Proliferative response was measured as the stimulation index (SI) = counts per minute (cpm) with antigen/cpm with no antigen. The % suppression = (cpm with candida minus cpm with brain fraction)/(cpm with candida – cpm with no antigen).

### 2.4 Flow Cytometry

The cells responding to antigen were isolated with flow cytometry. After 6 days of *in vitro* stimulation, dead cells were removed with density centrifugation, and the remaining cells were stained with PE-Cy7-anti-CD3, BV510-anti-CD4, PerCP-Cy5.5-anti-CD8, and FVS620 viability stain (all from Becton, Dickinson & Co., Franklin Lakes, NJ). A Becton Dickinson FACS Aria II flow cytometer was used to isolate the viable, CFSE-low (i.e., proliferating) CD3+ cells. Cells were sorted directly into Trizol (Thermo Fisher Scientific) for immediate lysis to preserve RNA and then frozen at −80°C. To verify acquisition of the desired population of cells, we did the initial sort into PBS rather than Trizol, and then ran the cells through the flow cytometer a second time. The sorted cells were consistently >90% viable, CFSE-low, and CD3+. A representative result from the flow cytometry is presented in **Supplementary Figure 2**.

### 2.5 RNA Extraction and RNA-Seq

Total RNA was extracted from the sorted cells with Takara NucleoSpin RNA XS columns and shipped to MedGenome (Foster City, CA) for library preparation and sequencing. The libraries for mRNA were prepared with the Takara SMARTer Stranded Total RNA-Seq kit v2-Pico Input Mammalian kit and sequenced on an Illumina NovaSeq system with paired-end, 150-base-pair reads. Libraries for TCR repertoire were prepared using the Takara SMARTer Human TCR a/b Profiling Kit and sequenced on the Illumina MiSeq system with paired end 300-base-pair reads.

### 2.6 Data Analysis

We performed statistical tests to identify differentially expressed genes (DEGs), adjusting for a Benjamini–Hochberg false discovery rate (FDR) of 0.05. Due to the low sample size, we applied three state-of-the-art methods, namely, edgeR (25), limma (26), and DESEq2 (27), and considered only genes that passed FDR of 0.05 in all three methods.

We used two types of comparisons to identify DEGs in the brain: (1) comparison between brain-stimulated and flu-stimulated PBMC from MS patients and (2) comparison between brain-stimulated and unstimulated PBMC from MS patients. Since the second type of comparison is between stimulated and unstimulated samples, we removed the effects of stimulation by excluding DEGs that were found also in comparisons of flu-stimulated and unstimulated PBMC and in brain-stimulated and unstimulated PBMC in the control group. Hierarchical clustering was performed using the Wald linkage. Gene Ontology (GO) enrichment was calculated using ToppGene (28). All data analyses were performed in Matlab R2019a.

## 3 Results

### 3.1 Brain Extraction and Tissue Culture Parameters

Our previous work on suppression of *in vitro* proliferation with brain homogenate was done with lymph node cells from mice immunized with ovalbumin in RPMI-1640 media with 10% FBS, using ovalbumin as the stimulating antigen and freshly prepared mouse brain homogenate for suppression (22). To define appropriate methods for human work, we tested stimulation and suppression of PBMC from 3 healthy controls using candida (can) as the stimulating antigen, various human brain fractions, and different tissue culture media. Results are given in **Table 1**. When using RPMI-1640 media supplemented with FBS, the results were consistent with our previous work. The crude brain homogenate (BH) strongly suppressed both the background proliferation with no antigen and the proliferation to candida. The denatured and extracted brain homogenate (Bd) had minimal effect on background (mean ± standard deviation change in cpm −327 ± 1,346) and modestly inhibited proliferation to candida (% suppression 24 ± 20 for Bd compared to 123 ± 28 for BH). The soluble proteins in the supernatant had minimal stimulatory or suppressive activity.

**Table 1:** Effects of tissue culture media on activity.

	AIM-V/human	RPMI-1640/FBS
**cpm**		
No Ag	338 ± 125	4,214 ± 2,201
supe	341 ± 131	
BH	838 ± 627	370 ± 146
Bd	846 ± 220	3,662 ± 1,637
can	29,631 ± 3,511	11,278 ± 1,649
**SI**		
supe	1.00 ± 0.01	
BH	2.23 ± 0.90	0.24 ± 0.20
Bd	2.73 ± 0.92	1.12 ± 0.63
can	78.1 ± 37.5	3.61 ± 1.86
can + BH	50.64 ± 42.0	0.93 ± 0.66
can + Bd	60.2 ± 3.2	2.82 ± 1.56
**% suppression**		
supe	13 ± 13	
BH	59 ± 18	123 ± 28
Bd	25 ± 2	24 ± 20

No Ag, no antigen; Can, candida; BH, brain homogenate; Bd, denatured brain homogenate; FBS, fetal bovine serum; supe, supernatant from initial homogenate. cpm is counts per minute; SI is stimulation index; SI and % suppression are defined in the methods.

Results were much different when using the same stimuli in AIM-V supplemented with autologous plasma. Background proliferation was lower, but proliferation to candida was higher. Both BH and Bd stimulated modest proliferation (SI 2.73 ± 0.92 for Bd). BH partially suppressed proliferation to candida, but the effect was less than in RPMI-1640/FBS. Bd also suppressed proliferation to candida, but to a lesser extent than BH. Subsequent investigation of three additional healthy subjects demonstrated that the difference was mainly due to the effects of FBS versus autologous plasma, rather than the media. Although proliferation with BH was similar to Bd, the proliferating cells were mostly not T cells. The mean ± sd number of CD3+, CFSE low cells sorted after BH stimulation was 7.8 ± 9.8% of the number sorted from Bd stimulated cells (*n* = 6). The remaining experiments described below were all done in AIM-V with autologous plasma using Bd as the antigen.

### 3.2 Proliferation

**Figure 1:**
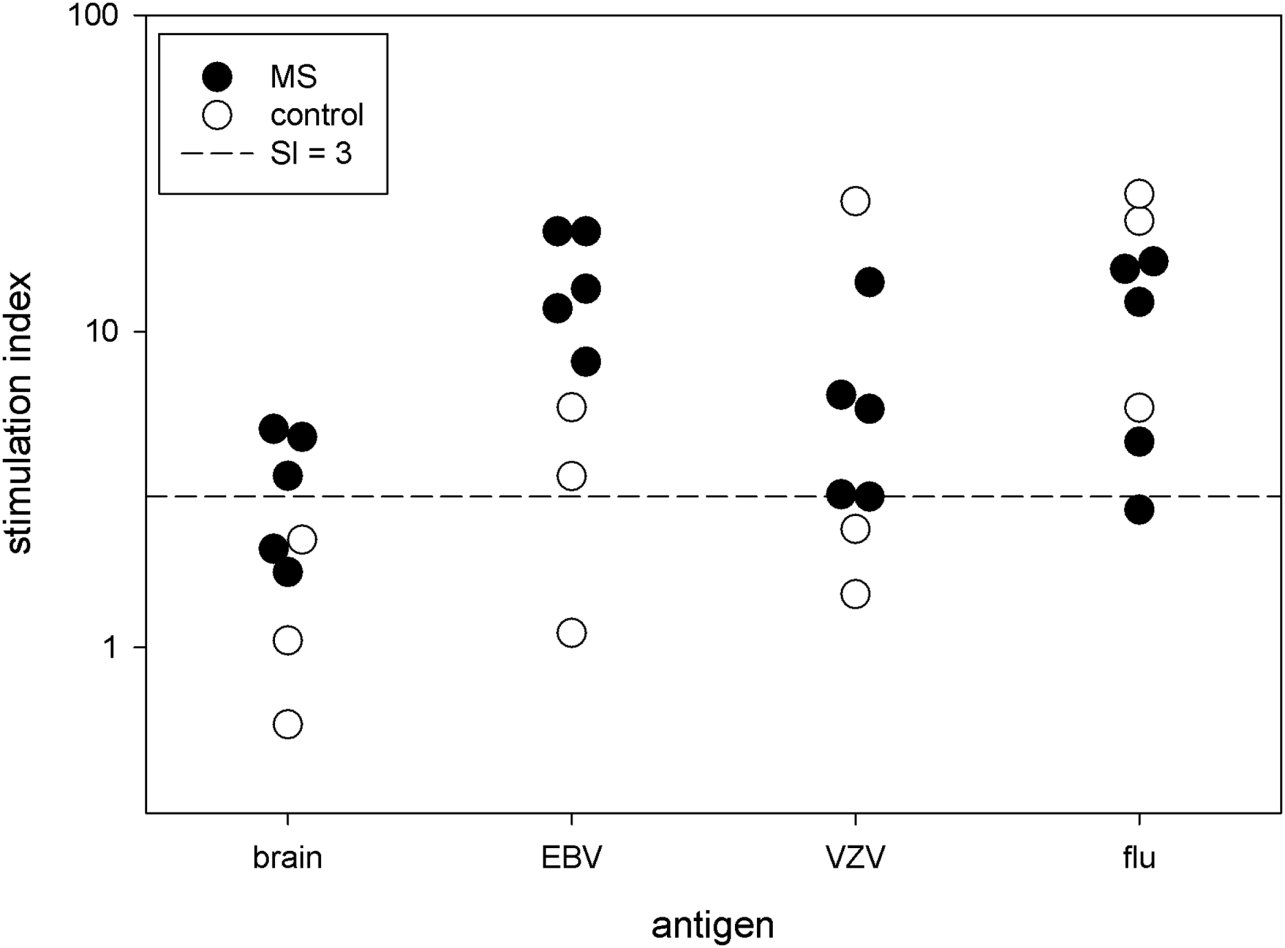
Proliferative response. PBMC from 5 MS patients and 3 controls were stimulated with brain antigens and 3 viruses. Each symbol denotes a single subject. The *y*-axis is the stimulation index.

We measured the proliferation to Bd in 5 MS patients and 3 controls. Brain antigens stimulated a modest proliferative response, which tended to be higher in the MS subjects than in the controls (**Figure 1**). The mean stimulation index for these 5 MS subjects is 3.4 ± 1.3. We tested proliferation to various viruses at the same time. These responses varied widely between subjects.

### 3.3 mRNA Expression

**Figure 2:**
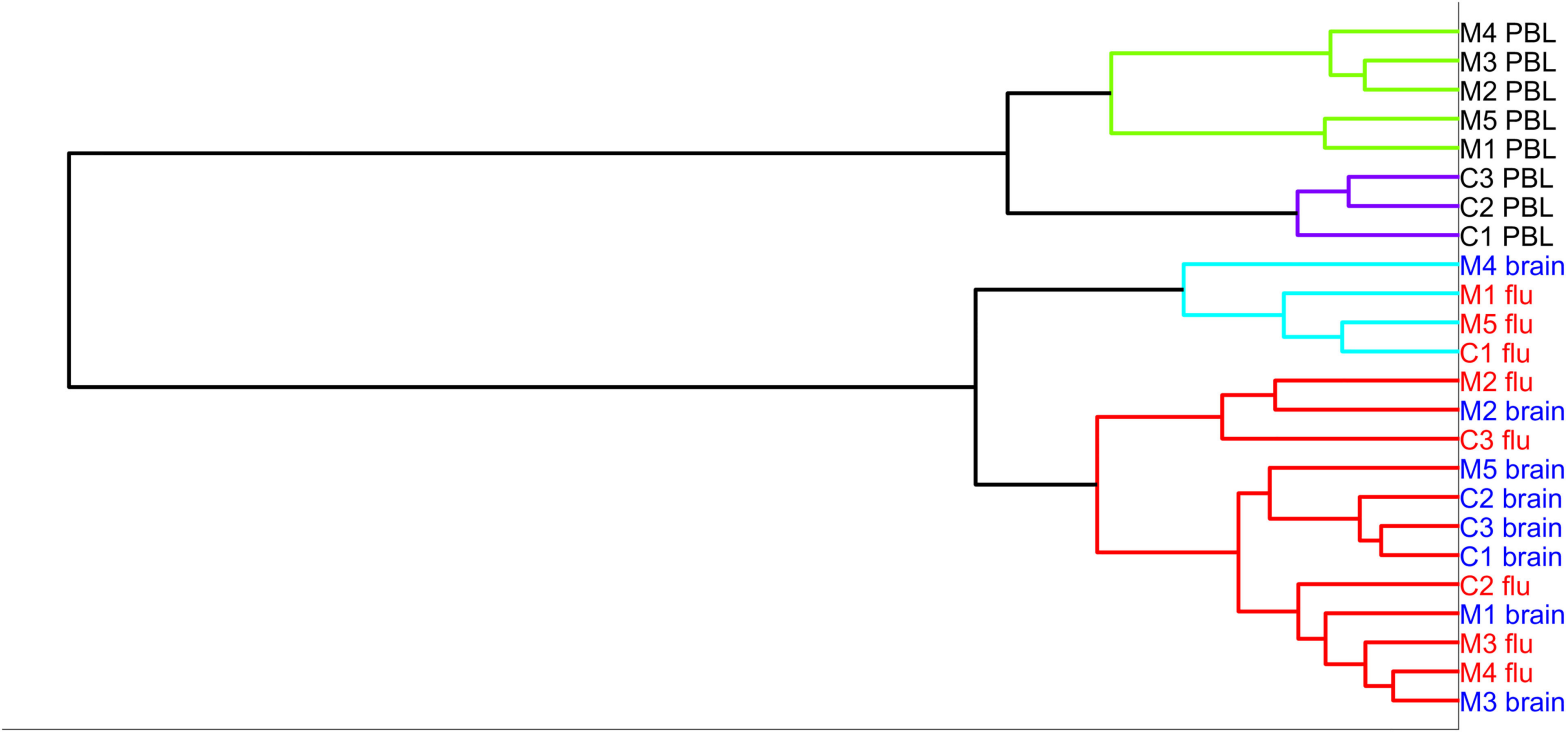
Hierarchical clustering of the five MS samples (MS)—brain-stimulated, flu-stimulated, and unstimulated, and three controls (C)—brain-stimulated, flu-stimulated, and unstimulated over the entire set of genes.

For these 8 subjects, we isolated the dividing T cells with flow cytometry. The number of T cells sorted following brain stimulation ranged from 2,100 to 72,400 (median 9,900 cells). The median number of sorted cells as a fraction of the number of cells initially cultured was 518 cells per million cultured cells. We extracted the RNA from the responding cells and sequenced the mRNA for cells stimulated with brain and flu. We also sequenced mRNA from unstimulated PBMC immediately *ex vivo*. Upon comparing transcriptomes with hierarchical clustering (**Figure 2**), the unstimulated samples from MS and controls all cluster together, and the control brain-stimulated samples are in one cluster with one of the MS brain-stimulated sample. The remaining 4 MS brain-stimulated samples are dispersed and often cluster with flu-stimulated samples. Principal component analysis gave similar results (**Supplementary Figure 2**).

### 3.4 Differentially Expressed Genes and Pathways

We tested 20,314 genes for differential expression between MS brain-stimulated and MS flu-stimulated samples. There were 16 DEGs that met the significance criteria for all 3 methods (EdgeR, DESeq2, and limma) (**Supplementary Table 1**). The expression of all of the 16 DEGs was higher with flu stimulation than with brain stimulation, and the majority are induced by interferon or have a known role in the antiviral response. The enrichment analysis with ToppGene found that the most enriched GO biological processes were type I interferon signaling and response to virus (**Table 2**).

**Table 2:** Enriched biological processes (B&H FDR < 0.05) for brain-stimulated vs. flu-stimulated MS blood cells.

ID	Name	FDR B&H	Genes from Input
GO:0060337	Type I interferon signaling pathway	E^-17^	10
GO:0071357, GO:0034340	Cellular response to type I interferon	E^-17^	10
GO:0009615	Response to virus	7E^-16^	12
GO:0051607	Defense response to virus	2E^-15^	11

There were 16 DEGs (**Supplementary Table 1**).

We also tested for differential expression between MS brain-stimulated and MS peripheral blood. There were 2,423 DEGs, which is expected since we are comparing cells proliferating after stimulation to unstimulated cells immediately *ex vivo*. To remove the cell growth or stimulation signal, we removed the genes that were also DEGs in the comparison of MS flu-stimulated versus MS blood. To make the results more MS specific, we also removed transcripts that were differentially expressed in the comparison of control brain versus control peripheral blood. This left 333 DEGs for pathway analysis (**Supplementary Table 2**). The most enriched GO biological processes were cytokine-mediated signaling, response to cytokine, and response to intereukin-1 (**Table 3**).

**Table 3:** Enriched biological processes (B&H FDR < 0.05) for brain-stimulated vs. unstimulated MS blood cells, excluding DEGs between flu-stimulated vs. unstimulated MS blood cells and between brain stimulated vs. unstimulated control blood cells.

ID	Name	FDR B&H	Genes from Input
GO:0019221	Cytokine-mediated signaling pathway	2E^-4^	36
GO:0034097	Response to cytokine	2E^-4^	47
GO:0071345	Cellular response to cytokine stimulus	8E^-4^	43
GO:0070555	Response to interleukin-1	0.01	14
GO:0051457	Maintenance of protein location in nucleus	0.05	5

There were 333 DEGs (**Supplementary Table 2**).

### 3.5 Expression of Individual mRNA

We also investigated changes in the expression of several individual transcripts of special interest for MS. These include transcription factors, cytokines, effector molecules, and adhesion/migration factors. The *p*-values given below are the maximal FDR-adjusted *p*-values using the combination of edgeR, limma, and DESEq2 methods. Statistical significance for all transcripts with all three methods are presented in **Supplementary Table 3**.

#### 3.5.1 Transcription Factors

**Figure 3:**
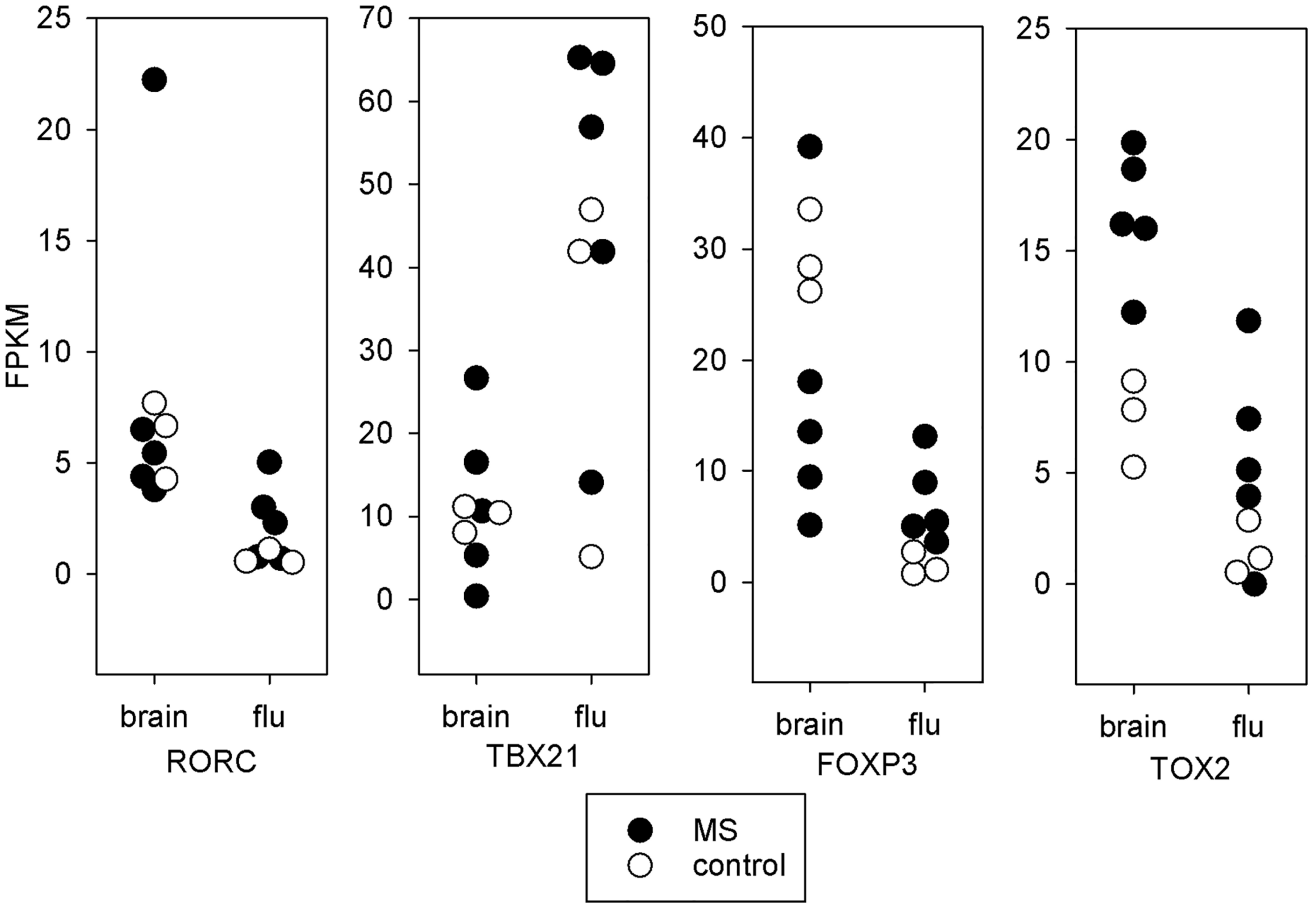
Transcription factors. mRNA expression in fragments per kilobase million (FPKM) for key T-cell transcription factors. Each point represents a single subject. Solid symbols are the 5 MS subjects and open symbols are the 3 controls. The brain stimulated cells from MS patients tend towards a Th17 or Tfh expression pattern. The brain stimulated cells from controls express increased FOXP3 relative to flu-stimulated cells. This is not seen in the MS patients.

In **Figure 3**, we present the expression levels for four transcription factors important for T-cell function. The RORC mRNA, which codes for the Th17 transcription factor RORγt, is part of 333 significant DEGs. RORC was 8.8-fold higher in MS brain-stimulated than MS unstimulated (FDR adjusted *p*-value < 0.04 across the three statistical tests). Similarly, RORC had a 5.5-fold change between control brain-stimulated and control unstimulated (FDR adjusted *p*-value < 0.001 in edgeR and limma, but insignificant in DESeq2). RORC is significantly increased in control brain-stimulated compared to control flu-stimulated (*p* < 0.04), but the difference did not reach significance for MS. In contrast, the TBX21 mRNA, which codes for the Th1 transcription factor T-bet, was increased only in flu-stimulated cells (for MS, 5-fold change, FDR-adjusted *p*-value < 0.005). FOXP3 mRNA, which codes for the Treg transcription factor Foxp3, was 6.4-fold higher in brain-stimulated cells compared to unstimulated cell in MS patients (adjusted *p*-value < 0.02), but the difference was more profound in the control brain-stimulated cells relative to either flu-stimulated cells (19-fold change, adjusted *p*-value < 0.0001) or unstimulated cells (37-fold change, *p* < 2e^-5^). Finally, mRNA for TOX2, a transcription factor linked to development of Tfh cells (29), was increased in MS brain-stimulated relative to unstimulated (24-fold change, *p* < 0.0001) and also in control brain-stimulated compared to unstimulated (11-fold change, *p* < 0.05). The higher expression of TOX2 in MS brain-stimulated relative to MS flu-stimulated or control brain-stimulated is noteworthy, but neither reaches statistical significance.

#### 3.5.2 Effector Molecules

**Figure 4:**
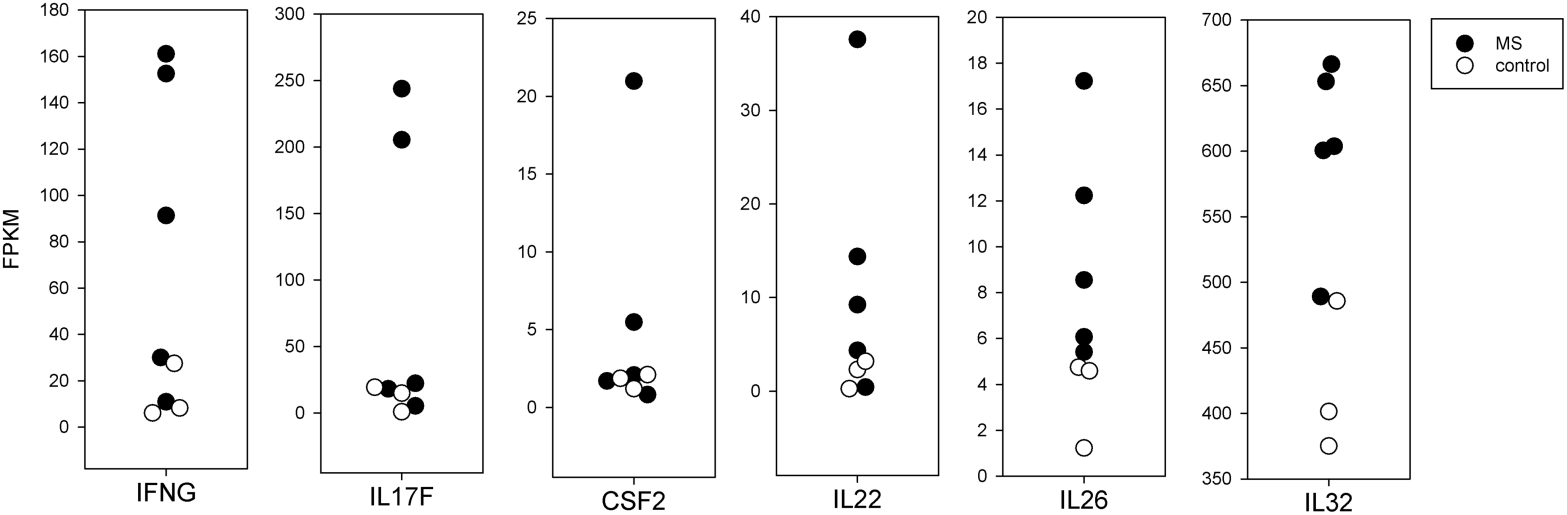
Expression of selected cytokine mRNA in brain-stimulated cells from MS and controls.

In **Figure 4**, we present the mRNA expression in brain-stimulated cells from MS compared to controls for several cytokines. For three cytokines long thought to be important in MS, IFNγ (IFNG), IL17F, and GM-CSF (CSF2), the values for MS and controls overlapped, but 2 or 3 MS subjects had markedly higher expression. These were from the subjects with higher SI in the proliferation assay. Other cytokines, including IL22, IL26, and IL32, were more consistently elevated in MS compared to controls, but these differences do not reach statistical significance.

#### 3.5.3 Adhesion/Migration

**Figure 5:**
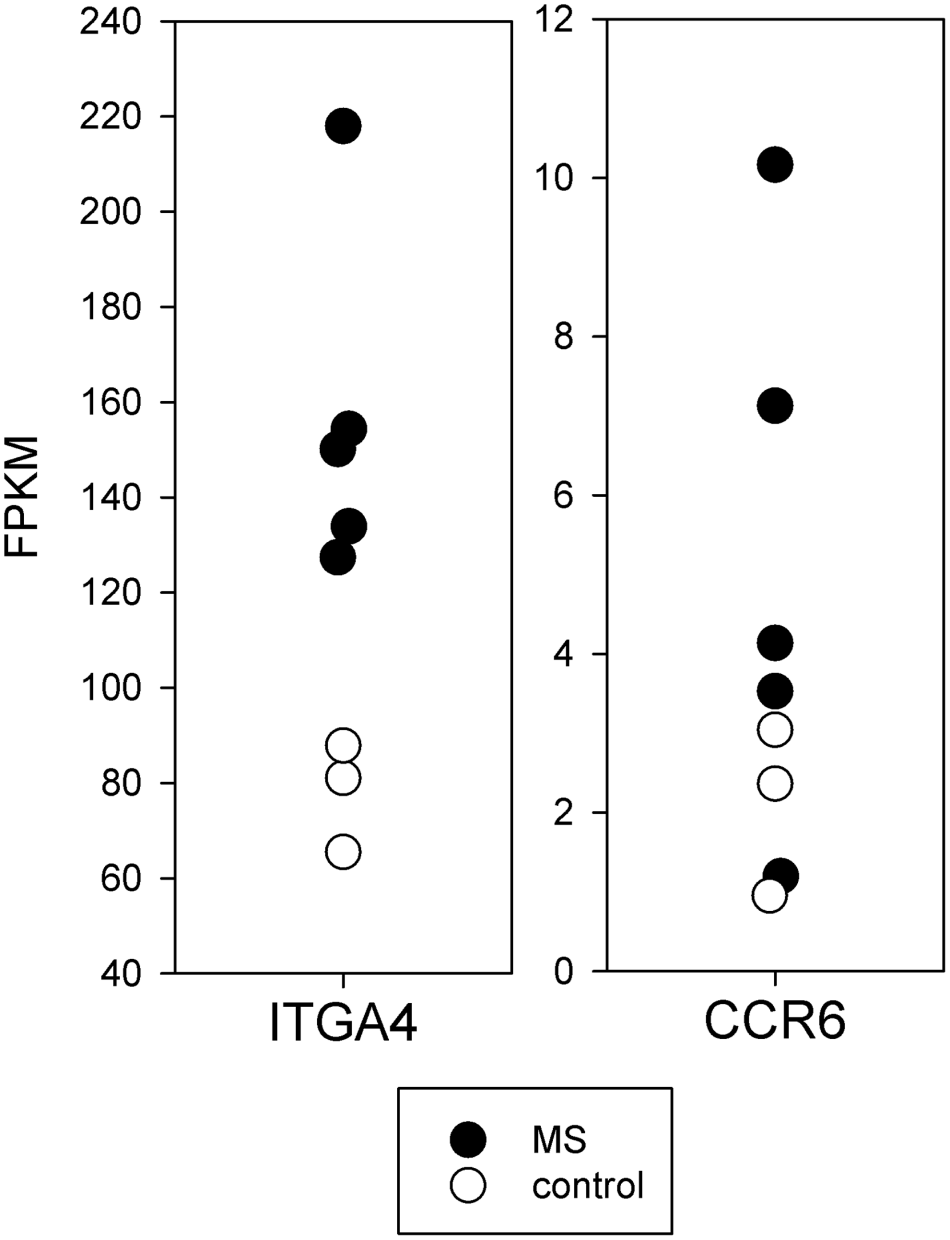
Expression of mRNA important for CNS entry in brain-stimulated cells from MS and controls. The mRNA for integrin-α4 required for T-cell entry into the CNS (ITGA4) was increased in MS (*p* < 0.01).

The mRNA for integrin-α4 required for T-cell entry into the CNS (30, 31) was increased in MS brain-stimulated compared to control brain-stimulated (*p* < 0.01) (**Figure 5**). The CCR6 chemokine receptor, also thought to be necessary for CNS entry, had a trend towards increased expression.

### 3.6 TCR Expression

We sequenced the TCR for cells stimulated with the various antigens and from the unstimulated PBMC, and analyzed the amino acid sequences of the CDR3 region of the beta chain.

#### 3.6.1 TCR Clonality

The clonality of the TCRβ CDR3 in the cells responding to brain stimulation is similar to the cells responding to EBV, VZV, or flu and higher than the unselected PBMC. The Simpson index and number of unique sequences are given in **Table 4**. This suggests that the cells proliferating in response to brain represent an antigen-specific response rather than non-specific proliferation.

**Table 4:** Simpson index for TCRβ CDR3 amino acid sequences.

Simpson index	Brain	EBV	VZV	Flu	PBMC
MS1	0.0202	0.0118	0.1376	0.0130	0.0012
MS2	0.0122	0.0056	0.0932	0.0198	0.0004
MS3	0.0103	0.0067	0.0068	0.0250	0.0001
MS4	0.0106	0.0047	0.0067	0.0091	0.0001
MS5	0.0714	0.0114	0.0345	0.0216	0.0007
**# unique sequences**	**Brain**	**EBV**	**VZV**	**Flu**	**PBMC**
MS1	201	628	106	866	57,312
MS2	372	913	205	343	103,579
MS3	789	1,219	985	136	25,611
MS4	229	2,019	409	1,168	22,888
MS5	783	1,277	776	845	173,887
**# total sequences**	**Brain**	**EBV**	**VZV**	**Flu**	**PBMC**
MS1	2,030	15,027	2,049	34,427	98,008
MS2	1,415	9,284	593	1,557	134,263
MS3	11,485	18,146	17,486	325	28,896
MS4	639	13,937	1,206	7,478	59,775
MS5	22,865	27,538	13,402	8,224	340,842

#### 3.6.2 Overlap of TCR With Different Antigens

**Figure 6:**
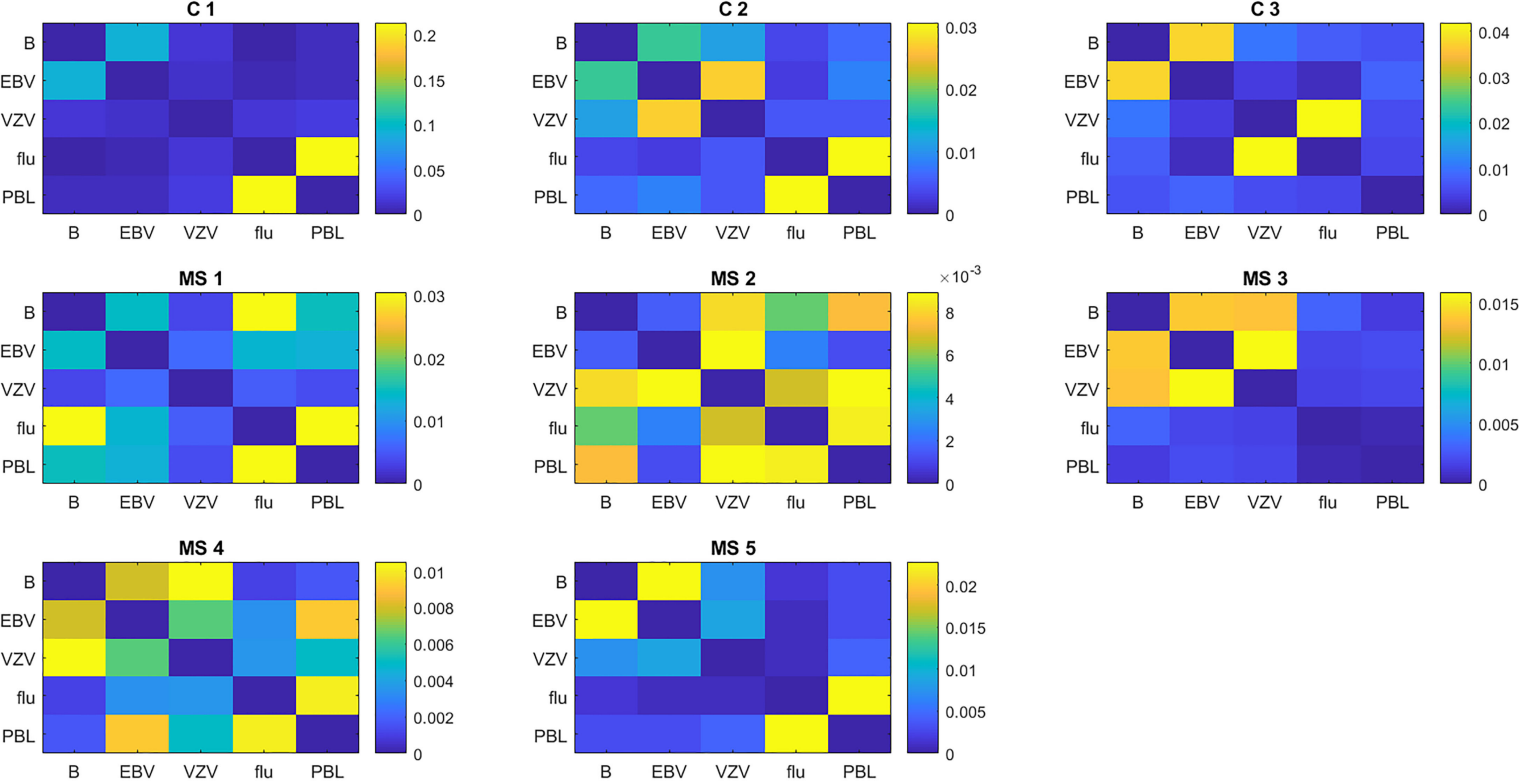
Similarity of the TCR repertoire to different antigens in individual subjects. Similarity is calculated as the weighted Jaccard index.

Molecular mimicry between brain antigens and EBV is a potential mechanism for the pathogenesis of MS (32, 33). To investigate this possibility, we calculated the similarity of TCR proliferating to various antigens using the weighted Jaccard index. In general, the TCR repertoire of the cells responding to any antigen had little similarity to the repertoire of cells from the same subject responding to other antigens (**Figure 6**). The degree of similarity varied widely between individuals, so we present the results for individual subjects. In the 3 controls, the brain-responding cells overlapped most with the EBV-responding cells. Brain-responding cells in the MS group could overlap with EBV, VZV, or flu. Also worth noting is that 2 of 3 controls and 4 of 5 MS patients have a high degree of overlap between flu-stimulated TCR and unselected PBMC TCR, suggesting that T cells specific for influenza are a major component of the circulating repertoire.

#### 3.6.3 TCR Shared Between Subjects

As expected, few TCR CDR3 sequences were shared between subjects. For the brain-stimulated cells, there were 5,166 unique sequences, and only 8 were found in more than one subject. Only one sequence, CASSLRHLNTEAFF, was common to two MS patients. This sequence was abundant in both, ranking 1/789 in one subject and 11/783 in the other.

## 4 Discussion

We are able to elicit a proliferative response to a complex mixture of brain antigens in PBMC from MS patients. The Bd fraction used for stimulation contains a broad range of proteins present in human brain. The epitopes that are processed and presented to T cells should resemble those present *in vivo*. Identification of the immunodominant epitopes in the mixture will be of great interest.

To our knowledge, this is the first report of successful use of a preparation containing a broad variety of brain proteins to stimulate at T-cell response. Very early similar efforts (34) used only the soluble fraction, which had little activity in our hands. More recent similar efforts (35) used a different measure for T-cell response and a commercial myelin extract, which is no longer available. We should note that the media used in tissue culture is critical, with FBS supplemented media producing much different results. We should also note that omission of the autologous plasma from AIM-V resulted in low numbers of sorted cells.

The magnitude of the proliferative response varies between subjects and is weaker than the response to common pathogens. However, the proliferation to Bd is more robust than that reported in the extensive literature on the T-cell response to MBP, PLP, or MOG. These studies often used SI > 2 as the criteria for a positive response (5, 36–39). A study of T-cell proliferation to peptides of aquaporin-4 in neuromyelitis optica patients also used SI > 2 as their cutoff value (40). They did not report the mean proliferation to their most stimulatory peptide, but we estimate that the SI is approximately 5 based on the published figure. This suggests that T-cell responses to self-antigens in autoimmune disease are not expected to be as vigorous as responses to infections.

With the mean SI around 3, the response is vigorous enough that we can select and sort dividing T cells in quantities sufficient to perform RNA-Seq. Few of the differences in mRNA expression between MS and controls are statistically significant, which is expected given the small number of samples, the variability between subjects, and the large number of comparisons. However, we can still reach some general conclusions. Transcription factor mRNA expression suggests that the brain-responding T cells in MS may have a Tfh phenotype. Integrin and chemokine receptor mRNA expression suggests that these cells have the ability to migrate to the CNS. We also support the recently reported relevance of interleukin-32 to MS (14, 15). Pathway analysis demonstrates that the activation of MS T cells with brain antigens results in a different response than stimulation with influenza, and a different response in MS than in controls.

The TCR repertoire in the brain-responsive cells had restricted diversity, as expected for an antigen-driven response. The brain-specific TCR in controls overlapped most with EBV, but the results in MS were more diverse, suggesting that the immune response to multiple viruses might stimulate T cells also able to recognize brain antigens.

These results are preliminary, and need to be amplified and extended. The findings need to be confirmed in larger numbers of subjects, and ideally, the MS patients should be studied early in disease before starting disease-modifying treatment. The mRNA findings need to be confirmed with immunoassays for cytokines and secreted proteins and flow cytometry for molecules expressed on the cell membrane. In addition, there are flaws that should be acknowledged. The brain antigen used for stimulation has been denatured, which has unknown effects on the repertoire of self-peptides that are processed and presented. Four of the five MS subjects were treated with ocrelizumab, which has a marked effect on disease activity and a potential effect on the *in vitro* T-cell responses.

In summary, we have demonstrated that we can stimulate proliferation to brain antigens in PBMC from MS and controls. The proliferating cells in MS patients have a pattern of mRNA expression suggesting pathogenic potential. These findings have several potentially useful applications. Analysis of the mRNA expression could define the pathogenic mechanisms in MS and suggest new targets for treatment of MS, such as interleukin-32. The response to brain antigens could define subtypes of MS useful for prognosis or selecting treatment. Analysis of the TCRs of the dominant clones could be used to identify the CNS antigen and define cross-reactive infections.

## Data Availability Statement

The data presented in the study are deposited in the Gene Expression Omnibus repository, accession number GSE193260.

## Ethics Statement

The studies involving human participants were reviewed and approved by Committee for the Protection of Human Subjects, University of Texas Health Science Center at Houston. The patients/participants provided their written informed consent to participate in this study.

## Author Contributions

AG contributed to data analysis, writing, and design of figures. HP performed much of the laboratory work and assisted with writing. JL conceived the study, assisted with data analysis, and wrote the manuscript. All authors contributed to the article and approved the submitted version.

## Funding

This work was funded in part by the Opal C. Rankin Professorship in Neurology.

## Conflict of Interest

The authors declare that the research was conducted in the absence of any commercial or financial relationships that could be construed as a potential conflict of interest.

## Publisher’s Note

All claims expressed in this article are solely those of the authors and do not necessarily represent those of their affiliated organizations, or those of the publisher, the editors and the reviewers. Any product that may be evaluated in this article, or claim that may be made by its manufacturer, is not guaranteed or endorsed by the publisher.
