# Supplementary material for: Brain Antigens Stimulate Proliferation of T Lymphocytes With a Pathogenic Phenotype in Multiple Sclerosis Patients
Source: Front Immunol. 2022 Jan 31;13:835763. doi: 10.3389/fimmu.2022.835763 (PMC8841344; doi:10.3389/fimmu.2022.835763)
Supplement: Supplementary file 1 [file Image_1.pdf]

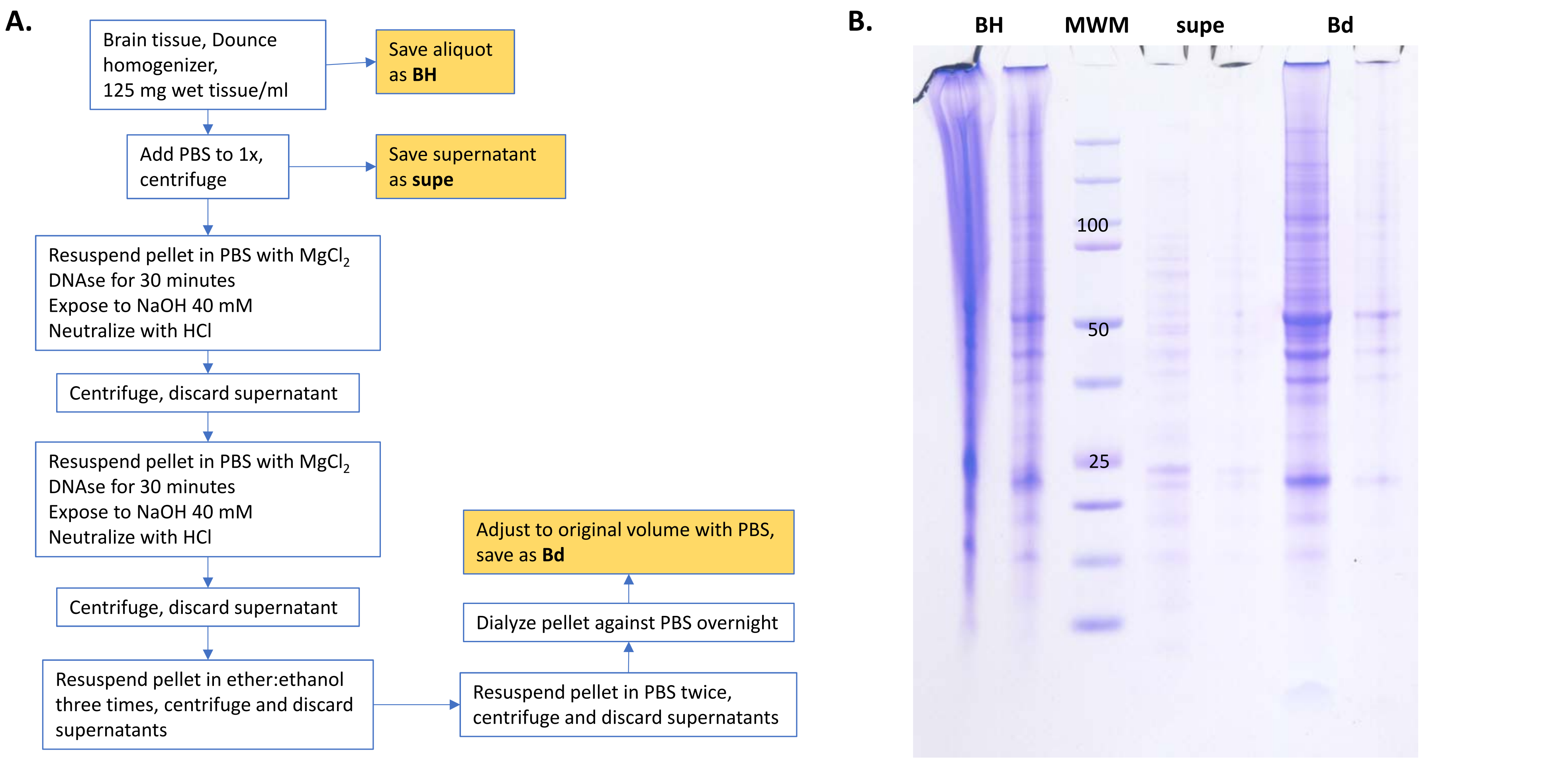


**Supplementary Figure 1:** A. Schematic of procedure. All centrifugations were done at 16,000g for 5 minutes.
B. Coomassie stained gel of fractions BH, supe, and Bd; each fraction is run at two concentrations, 5 µl/lane and 1 µl/lane. BH does not run well on the gel, but comparison of BH 1 µl/lane with Bd demonstrates the presence of similar major protein bands. The lane labeled MWM is molecular weight markers, with selected molecular weights indicated.
