# Supplementary material for: Brain Antigens Stimulate Proliferation of T Lymphocytes With a Pathogenic Phenotype in Multiple Sclerosis Patients
Source: Front Immunol. 2022 Jan 31;13:835763. doi: 10.3389/fimmu.2022.835763 (PMC8841344; doi:10.3389/fimmu.2022.835763)
Supplement: Supplementary file 2 [file Image_2.pdf]

BD FACSDiva 8.0.1

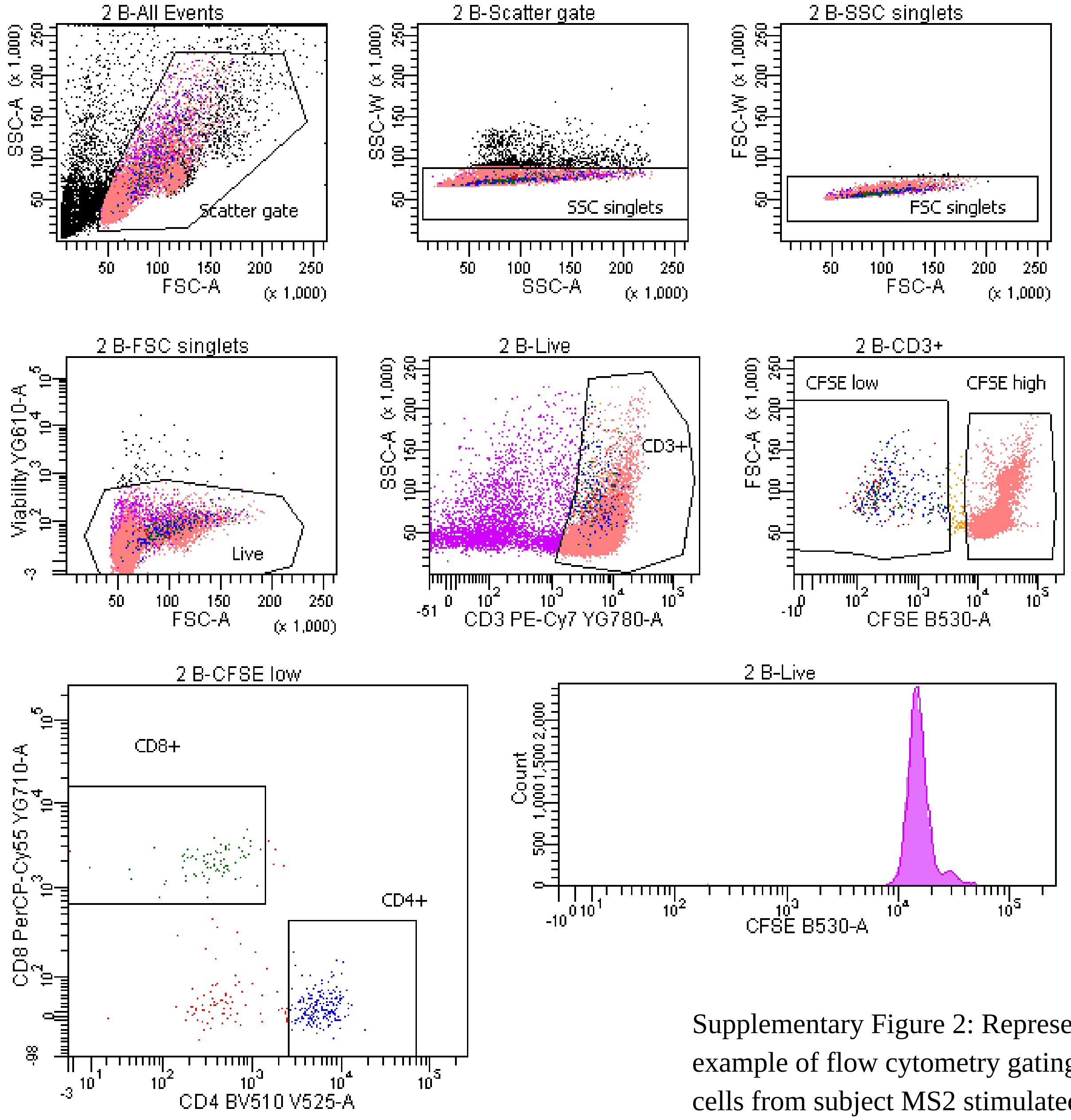

Tube: 2 B

Population	#Events	%Parent	%Total
All Events	37,083	####	100.0
Scatter gate	31,509	85.0	85.0
SSC singlets	30,269	96.1	81.6
FSC singlets	30,260	100.0	81.6
Live	30,183	99.7	81.4
CD3+	26,537	87.9	71.6
CFSE high	26,091	98.3	70.4
CFSE low	370	1.4	1.0
CD4+	206	55.7	0.6
CD8+	80	21.6	0.2

Supplementary Figure 2: Representative example of flow cytometry gating. These are cells from subject MS2 stimulated with Bd. Forward and side scatter are used to gate on single cells, which are subsequently gated for viability, CD3+, and CFSE low. CD4 and CD8 were included for interest, but not used for sorting. All viable, CD3+, CFSE low cells were sorted for RNA extraction.
