# Supplementary material for: Brain Antigens Stimulate Proliferation of T Lymphocytes With a Pathogenic Phenotype in Multiple Sclerosis Patients
Source: Front Immunol. 2022 Jan 31;13:835763. doi: 10.3389/fimmu.2022.835763 (PMC8841344; doi:10.3389/fimmu.2022.835763)
Supplement: Supplementary file 3 [file Image_3.pdf]

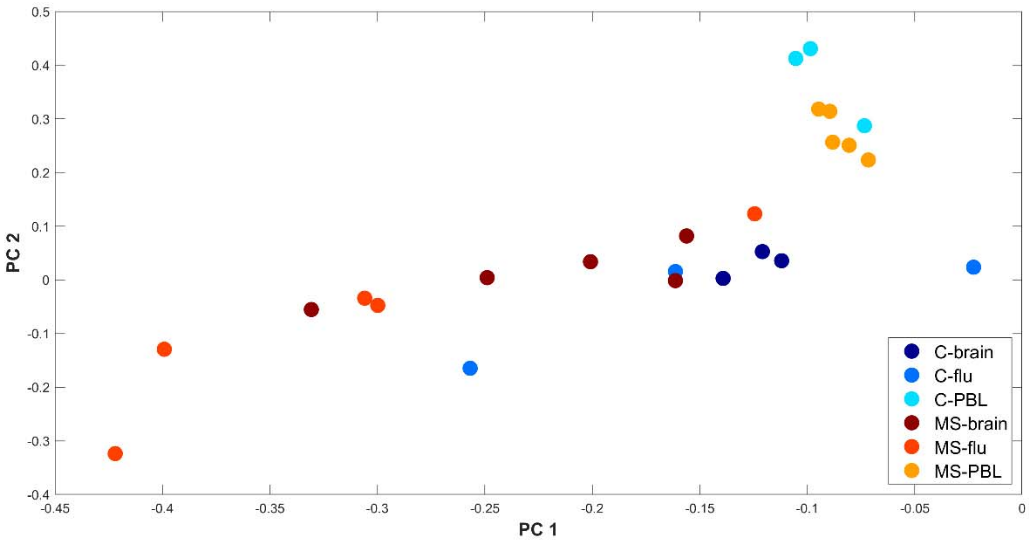

Supplementary Figure 3: Principal Component Analysis plot of the five MS samples (MS) – brain-stimulated, flu-stimulated and unstimulated and 3 control samples (C) – brain-stimulated, flu-stimulated and unstimulated over the entire set of genes. The unstimulated samples are separate from the stimulated samples. Additionally, the control samples stimulated by brain form a tight cluster.
