# Supplementary material for: Brain Antigens Stimulate Proliferation of T Lymphocytes With a Pathogenic Phenotype in Multiple Sclerosis Patients
Source: Front Immunol. 2022 Jan 31;13:835763. doi: 10.3389/fimmu.2022.835763 (PMC8841344; doi:10.3389/fimmu.2022.835763)
Supplement: Supplementary file 4 [file Table_1.pdf]

Significant DEGs, MS brain vs MS flu	q-value edgeR	q-value limma	q-value DESeq2	Fold Change
CMPK2	0.0014	0.0037	2.E-09	0.08
HELZ2	0.0036	0.0037	1.E-07	0.06
IFI27	0.0005	0.0003	4.E-11	0.00
IFI44L	0.0040	0.0003	4.E-07	0.01
IFI6	0.0005	0.0027	3.E-12	0.06
IFIT1	0.0014	0.0003	2.E-08	0.00
IFIT3	0.0110	0.0463	4.E-07	0.06
ISG15	0.0040	0.0118	9.E-10	0.05
LGALS9	0.0395	0.0463	5.E-05	0.22
LY6E	0.0036	0.0112	3.E-08	0.17
MX1	0.0005	0.0003	7.E-17	0.04
OAS1	0.0061	0.0118	2.E-09	0.05
OAS3	0.0395	0.0220	2.E-06	0.16
PLSCR1	0.0389	0.0276	7.E-06	0.25
RSAD2	0.0242	0.0027	1.E-05	0.04
USP18	0.0022	0.0003	6.E-06	0.03
