# Supplementary material for: Brain Antigens Stimulate Proliferation of T Lymphocytes With a Pathogenic Phenotype in Multiple Sclerosis Patients
Source: Front Immunol. 2022 Jan 31;13:835763. doi: 10.3389/fimmu.2022.835763 (PMC8841344; doi:10.3389/fimmu.2022.835763)
Supplement: Supplementary file 5 [file Table_2.pdf]

Significant DEGs, MS brain vs MS PBL (* excluding singificant MS flu vs MS PBL and Control brain vs. control PBL)	q-value edgeR	q-value limma	q-value DESeq2	Fold change
AAGAB	0.0082	0.0077	0.0283	4.0
ABCF1	0.0134	0.0110	0.0132	3.4
ABHD17A	0.0030	0.0144	0.0115	0.9
ADAP1	0.0031	0.0019	0.0002	0.2
AGPAT9	0.0191	0.0085	0.0275	0.2
AHI1	0.0052	0.0061	0.0019	7.7
AIG1	0.0009	0.0006	0.0364	0.2
AKAP8L	0.0029	0.0026	0.0057	0.7
AKNA	0.0224	0.0161	0.0049	0.9
AMD1	0.0216	0.0049	0.0264	2.6
AMY2B	0.0017	0.0008	0.0050	0.3
ANKRD28	0.0241	0.0089	0.0349	3.0
ANAPC15	0.0272	0.0324	0.0213	3.7
AP5B1	0.0203	0.0147	0.0219	0.4
APITD1-CORT	0.0207	0.0229	0.0450	94.0
APOA1BP	0.0267	0.0146	0.0439	4.1
ARFGEF1	0.0115	0.0090	0.0155	0.7
ARGLU1	0.0339	0.0421	0.0276	1.0
ARHGAP11B	0.0120	0.0062	0.0160	6.6
ARHGAP25	0.0226	0.0210	0.0421	0.9
ARHGAP9	0.0051	0.0105	0.0042	0.8
ARL15	0.0349	0.0189	0.0346	0.4
ARSD	0.0012	0.0007	0.0390	0.1
ARSG	0.0019	0.0011	0.0024	0.2
ASCL2	0.0031	0.0023	0.0427	0.1
ASGR1	0.0018	0.0015	0.0272	0.2
ASNSD1	0.0105	0.0204	0.0061	5.2
ASUN	0.0024	0.0028	0.0119	5.1
ATAD5	0.0007	0.0001	0.0138	9.5
ATHL1	0.0111	0.0033	0.0007	0.2
ATP2A2	0.0365	0.0149	0.0401	3.1
BCL2L13	0.0227	0.0153	0.0437	0.7
BRCA2	0.0105	0.0016	0.0135	16.9
BTG3	0.0170	0.0183	0.0321	4.4
BUB3	0.0350	0.0078	0.0384	3.0
C10orf11	0.0022	0.0000	0.0001	0.0
C11orf82	0.0220	0.0075	0.0264	14.6
C12orf57	0.0184	0.0465	0.0484	0.8
C14orf166	0.0044	0.0061	0.0026	3.9
C16orf54	0.0342	0.0095	0.0219	0.6
C19orf40	0.0075	0.0030	0.0198	11.8

C1RL	0.0077	0.0046	0.0220	0.4
C21orf91	0.0050	0.0027	0.0092	3.7
C3orf62	0.0119	0.0087	0.0190	0.4
C9orf156	0.0029	0.0064	0.0074	0.6
CALD1	0.0020	0.0004	0.0120	0.0
CAMKK2	0.0084	0.0052	0.0304	0.4
CAMKMT	0.0247	0.0134	0.0446	0.4
CAND1	0.0317	0.0108	0.0142	3.1
CANX	0.0115	0.0061	0.0072	2.9
CASP6	0.0024	0.0047	0.0019	7.7
CBFB	0.0331	0.0476	0.0119	4.9
CCDC117	0.0030	0.0067	0.0014	7.6
CCDC130	0.0166	0.0084	0.0114	0.4
CCDC25	0.0243	0.0040	0.0242	3.1
CCDC57	0.0108	0.0140	0.0388	0.7
CCDC88B	0.0015	0.0058	0.0219	0.8
CCL22	0.0149	0.0008	0.0262	Inf
CCL3L3	0.0019	0.0067	0.0001	0.0
CCR1	0.0028	0.0044	0.0001	0.2
CCT2	0.0496	0.0184	0.0443	3.0
CCT6A	0.0020	0.0008	0.0001	5.4
CD101	0.0013	0.0008	0.0205	0.2
CD8B	0.0160	0.0064	0.0040	0.3
CDC23	0.0168	0.0097	0.0437	4.5
CDC27	0.0001	0.0000	0.0000	5.6
CDC42SE1	0.0046	0.0063	0.0017	0.9
CDYL2	0.0184	0.0384	0.0085	5.7
CISD2	0.0122	0.0192	0.0232	5.9
CISH	0.0492	0.0255	0.0432	0.4
CKAP2	0.0010	0.0011	0.0005	5.2
CLIC5	0.0031	0.0004	0.0263	15.1
CLTB	0.0178	0.0167	0.0238	3.3
COL18A1	0.0015	0.0004	0.0212	0.1
COMMD9	0.0157	0.0177	0.0471	0.7
CREBZF	0.0267	0.0165	0.0452	0.8
CTBP2	0.0018	0.0009	0.0002	0.2
CTC-429P9.4	0.0129	0.0100	0.0014	159.0
CTC-487M23.8	0.0028	0.0023	0.0024	7.6
CTDSP1	0.0060	0.0160	0.0377	0.8
CYBA	0.0026	0.0115	0.0021	0.9
CYFIP1	0.0162	0.0131	0.0312	0.4
DBF4	0.0120	0.0257	0.0062	5.2
DCAF13	0.0025	0.0007	0.0059	5.4
DCLRE1A	0.0174	0.0012	0.0246	7.7
DDA1	0.0259	0.0431	0.0254	5.9
DDB1	0.0128	0.0024	0.0044	2.9
DENND4B	0.0267	0.0244	0.0274	0.7

DGCR14	0.0103	0.0067	0.0202	0.5
DGCR2	0.0142	0.0093	0.0128	0.4
DGKI	0.0124	0.0015	0.0127	82.0
DGKZ	0.0128	0.0096	0.0102	0.7
DKC1	0.0080	0.0168	0.0016	5.7
DLAT	0.0077	0.0046	0.0281	6.8
DOPEY2	0.0095	0.0048	0.0450	0.4
EBI3	0.0170	0.0042	0.0350	99.5
EDAR	0.0010	0.0001	0.0460	0.0
EGR1	0.0057	0.0294	0.0002	0.4
EIF4E	0.0004	0.0008	0.0001	5.3
EIF4G2	0.0128	0.0023	0.0006	2.7
ELOVL5	0.0260	0.0350	0.0177	3.7
EPHB6	0.0022	0.0021	0.0133	0.3
ERC1	0.0010	0.0014	0.0020	0.5
ERO1LB	0.0018	0.0018	0.0350	0.4
ETF1	0.0063	0.0057	0.0066	3.5
EVL	0.0057	0.0137	0.0024	0.9
FAIM3	0.0100	0.0031	0.0003	0.3
FAM104A	0.0071	0.0054	0.0157	3.5
FAM107B	0.0095	0.0021	0.0001	3.0
FAM110A	0.0087	0.0092	0.0363	0.6
FAM160A2	0.0013	0.0010	0.0161	0.3
FAM26F	0.0002	0.0000	0.0001	0.0
FAM64A	0.0004	0.0000	0.0010	70.6
FCER2	0.0057	0.0040	0.0334	0.0
FGD3	0.0015	0.0010	0.0001	0.4
FLOT2	0.0032	0.0130	0.0148	0.9
FLT3LG	0.0067	0.0082	0.0087	0.5
GALNS	0.0359	0.0208	0.0410	0.5
GATAD1	0.0033	0.0019	0.0493	0.4
GDI2	0.0143	0.0068	0.0085	2.5
GEN1	0.0314	0.0184	0.0093	12.6
GIMAP1	0.0039	0.0020	0.0043	0.3
GIMAP2	0.0472	0.0165	0.0395	0.6
GIMAP5	0.0423	0.0446	0.0398	0.9
GLI4	0.0027	0.0019	0.0030	0.2
GLIPR1	0.0115	0.0051	0.0090	0.5
GMIP	0.0027	0.0056	0.0110	0.8
GNG4	0.0251	0.0031	0.0233	Inf
GNL2	0.0300	0.0127	0.0351	3.2
GNPTG	0.0136	0.0085	0.0381	0.3
GNA15	0.0041	0.0133	0.0040	6.5
GPHN	0.0090	0.0020	0.0407	4.0
GPRIN3	0.0096	0.0022	0.0041	3.7
GPSM2	0.0072	0.0011	0.0381	11.2
GPSM3	0.0220	0.0169	0.0066	0.8

GRK6	0.0030	0.0030	0.0021	0.6
GSTK1	0.0238	0.0204	0.0127	0.8
GTDC1	0.0154	0.0214	0.0255	4.0
GTF2E2	0.0133	0.0082	0.0159	3.7
GTSF1	0.0132	0.0126	0.0312	9.6
GZMH	0.0103	0.0188	0.0085	0.4
H6PD	0.0119	0.0067	0.0497	0.5
HADH	0.0206	0.0355	0.0202	7.0
HAGH	0.0220	0.0157	0.0206	0.4
HAL	0.0002	0.0001	0.0142	0.0
HDAC2	0.0037	0.0018	0.0041	3.4
HDAC3	0.0070	0.0060	0.0124	3.7
HDAC4	0.0304	0.0144	0.0238	0.6
HDLBP	0.0175	0.0144	0.0115	3.1
HLA-A	0.0115	0.0244	0.0029	1.0
HMGN1	0.0011	0.0009	0.0001	3.5
HNRNPA3	0.0342	0.0036	0.0116	2.4
HNRNPR	0.0087	0.0020	0.0008	3.0
HPGD	0.0027	0.0019	0.0174	6.2
HSPA4	0.0094	0.0097	0.0016	5.2
ICAM3	0.0024	0.0044	0.0030	0.7
IFFO1	0.0128	0.0073	0.0186	0.4
IFIT2	0.0007	0.0005	0.0208	0.1
IL17A	0.0145	0.0005	0.0010	Inf
IL22	0.0146	0.0009	0.0008	Inf
IL26	0.0003	0.0000	0.0002	494.0
IL4I1	0.0009	0.0001	0.0220	12.2
ING1	0.0124	0.0142	0.0174	0.5
INPP1	0.0055	0.0011	0.0165	6.0
INPP4A	0.0172	0.0181	0.0450	0.8
IPO7	0.0032	0.0071	0.0012	6.8
IRF5	0.0056	0.0125	0.0262	0.5
ITGAE	0.0001	0.0001	0.0000	6.8
ITIH4	0.0033	0.0025	0.0311	0.2
KDM4B	0.0163	0.0139	0.0353	0.4
KIAA1217	0.0037	0.0005	0.0155	123.0
KIF20B	0.0006	0.0006	0.0031	8.5
KIF3A	0.0223	0.0187	0.0439	4.6
KTN1	0.0149	0.0113	0.0084	3.7
LCOR	0.0032	0.0037	0.0056	0.5
LGALS9	0.0078	0.0178	0.0393	0.7
LHPP	0.0137	0.0083	0.0282	0.4
LMF1	0.0024	0.0014	0.0104	0.2
LRP8	0.0031	0.0009	0.0203	5.6
LY75-CD302	0.0004	0.0003	0.0274	0.0
MAL	0.0085	0.0130	0.0157	0.6
MAP4	0.0164	0.0099	0.0449	3.0

MAST3	0.0013	0.0012	0.0177	0.4
MBNL2	0.0046	0.0032	0.0371	0.5
MGLL	0.0061	0.0072	0.0029	0.2
MINOS1	0.0098	0.0168	0.0073	4.7
MLH1	0.0011	0.0031	0.0002	7.9
MMADHC	0.0031	0.0017	0.0052	3.7
MSRB1	0.0339	0.0331	0.0209	0.4
MT1F	0.0110	0.0173	0.0013	26.2
MX2	0.0009	0.0003	0.0000	0.3
MYD88	0.0115	0.0098	0.0057	0.6
MYH10	0.0045	0.0037	0.0002	35.1
MYO18A	0.0254	0.0266	0.0362	0.9
NCF4	0.0214	0.0151	0.0341	0.4
NDUFB4	0.0264	0.0328	0.0349	3.8
NDUFV2	0.0159	0.0094	0.0080	3.3
NIFK	0.0016	0.0021	0.0004	3.7
NKTR	0.0093	0.0045	0.0004	0.5
NPIPA7	0.0489	0.0667	0.2386	0.9
NR2C2	0.0230	0.0215	0.0335	0.7
NUDCD1	0.0020	0.0012	0.0154	7.1
NUP50	0.0164	0.0078	0.0080	2.8
NUP54	0.0035	0.0023	0.0148	3.7
NXPE3	0.0097	0.0055	0.0406	0.5
NADK	0.0048	0.0052	0.0062	0.7
NAT9	0.0012	0.0015	0.0168	0.5
OLA1	0.0067	0.0148	0.0013	5.4
OSTC	0.0083	0.0028	0.0154	3.3
PACS1	0.0110	0.0117	0.0105	0.7
PACS2	0.0159	0.0137	0.0077	0.4
PARP10	0.0233	0.0204	0.0275	0.7
PATL2	0.0084	0.0038	0.0226	0.5
PCED1A	0.0145	0.0056	0.0113	0.3
PCED1B	0.0122	0.0135	0.0092	0.5
PDZD11	0.0046	0.0100	0.0063	8.7
PERP	0.0053	0.0010	0.0385	17.7
PHF20	0.0038	0.0046	0.0349	0.7
PIGL	0.0162	0.0096	0.0062	0.4
PIK3CD	0.0168	0.0226	0.0382	0.9
PIP5K1C	0.0119	0.0087	0.0447	0.6
PLEKHB1	0.0068	0.0049	0.0421	0.2
PLXNC1	0.0478	0.0160	0.0444	0.5
POGZ	0.0087	0.0078	0.0139	0.8
POLE3	0.0134	0.0191	0.0118	3.7
PPARG	0.0066	0.0025	0.0204	14.6
PPP1CC	0.0500	0.0334	0.0214	3.0
PPP1R3E	0.0089	0.0056	0.0358	0.3
PPP2R1B	0.0196	0.0187	0.0243	5.3

PRKCB	0.0023	0.0118	0.0039	0.7
PRPF38B	0.0343	0.0317	0.0423	0.9
PSMA1	0.0024	0.0025	0.0025	3.2
PSMD11	0.0053	0.0025	0.0150	3.6
PSMD12	0.0437	0.0215	0.0373	3.1
PSTPIP1	0.0199	0.0370	0.0334	0.9
PTEN	0.0143	0.0064	0.0068	0.6
PTGER2	0.0458	0.0276	0.0415	0.5
PTGES3	0.0058	0.0033	0.0012	3.0
PTPN18	0.0029	0.0045	0.0007	0.7
PUS7	0.0058	0.0056	0.0205	6.5
PYCARD	0.0009	0.0034	0.0003	0.6
RAB11FIP1	0.0095	0.0242	0.0191	0.7
RAP1GAP2	0.0063	0.0045	0.0139	0.3
RASGRP4	0.0014	0.0019	0.0031	0.3
RASSF1	0.0357	0.0209	0.0350	0.6
RBM14-RBM4	0.0000	0.0000	0.0001	0.0
RBPJ	0.0017	0.0007	0.0002	3.5
RDX	0.0059	0.0162	0.0008	6.9
RELB	0.0300	0.0124	0.0437	3.4
RGS19	0.0048	0.0061	0.0042	0.7
RIC3	0.0037	0.0026	0.0131	0.2
RIN1	0.0027	0.0016	0.0393	0.1
RNPC3	0.0151	0.0050	0.0078	0.3
RNPEPL1	0.0191	0.0105	0.0210	0.6
RORC	0.0295	0.0127	0.0353	8.8
RP11-977G19.10	0.0057	0.0177	0.0006	8.9
RRP1B	0.0115	0.0055	0.0370	3.5
SACS	0.0356	0.0214	0.0478	4.2
SAMD3	0.0234	0.0118	0.0082	0.5
SBK1	0.0024	0.0015	0.0094	0.2
SENP7	0.0254	0.0085	0.0462	0.6
SET	0.0005	0.0002	0.0000	3.6
SH2B2	0.0005	0.0006	0.0386	0.1
SIGIRR	0.0100	0.0077	0.0042	0.6
SIN3B	0.0077	0.0087	0.0485	0.6
SKP1	0.0031	0.0017	0.0003	3.0
SLC25A28	0.0457	0.0146	0.0416	0.5
SLC39A8	0.0162	0.0084	0.0336	4.6
SLC9B2	0.0140	0.0064	0.0170	5.3
SLCO3A1	0.0095	0.0166	0.0221	0.6
SMARCE1	0.0477	0.0089	0.0320	2.3
SMCR8	0.0121	0.0076	0.0119	0.6
SNRPA1	0.0294	0.0494	0.0272	4.3
SP100	0.0326	0.0228	0.0206	0.8
SP110	0.0225	0.0120	0.0077	0.8
SPSB3	0.0021	0.0021	0.0016	0.6

SRP19	0.0072	0.0055	0.0273	3.8
SRP9	0.0096	0.0047	0.0054	3.2
SRPK1	0.0063	0.0015	0.0024	4.0
SRSF10	0.0203	0.0038	0.0035	2.6
STAT2	0.0002	0.0002	0.0000	0.5
STRIP2	0.0155	0.0067	0.0020	59.8
STX10	0.0250	0.0292	0.0341	0.7
SUB1	0.0031	0.0019	0.0001	3.8
SUMO1	0.0013	0.0018	0.0004	4.4
SUN1	0.0043	0.0043	0.0319	0.6
SUN2	0.0033	0.0021	0.0001	0.7
TAF9	0.0035	0.0020	0.0006	4.3
TAGLN	0.0056	0.0054	0.0426	0.1
TANC2	0.0005	0.0002	0.0002	0.2
TBC1D22A	0.0028	0.0073	0.0323	0.7
TEP1	0.0153	0.0122	0.0107	0.4
TFIP11	0.0322	0.0436	0.0230	4.3
TIMM17A	0.0109	0.0106	0.0159	5.6
TLE2	0.0046	0.0024	0.0477	0.2
TLK1	0.0327	0.0213	0.0101	0.7
TMEM140	0.0065	0.0073	0.0189	0.6
TMEM164	0.0365	0.0270	0.0407	0.5
TMEM237	0.0092	0.0128	0.0045	14.7
TMEM55A	0.0044	0.0031	0.0200	0.4
TMEM71	0.0153	0.0039	0.0140	0.5
TNRC6C	0.0070	0.0049	0.0184	0.6
TOMM7	0.0186	0.0167	0.0189	0.8
TPK1	0.0025	0.0032	0.0291	0.5
TRAF3IP3	0.0122	0.0119	0.0062	0.8
TRIM3	0.0029	0.0042	0.0174	0.4
TRIM38	0.0023	0.0017	0.0126	0.6
TRMT6	0.0222	0.0316	0.0296	4.1
TSPAN32	0.0003	0.0001	0.0007	0.0
TSPO	0.0005	0.0021	0.0009	0.5
TTC1	0.0291	0.0165	0.0478	3.1
TTF1	0.0039	0.0107	0.0011	8.4
TYMP	0.0004	0.0012	0.0001	0.5
UBA7	0.0035	0.0012	0.0003	0.5
UBE2G1	0.0367	0.0085	0.0279	2.8
UBE2L3	0.0262	0.0331	0.0480	3.9
UBQLN2	0.0086	0.0072	0.0358	0.7
UNKL	0.0183	0.0084	0.0105	0.4
URM1	0.0170	0.0117	0.0346	3.6
USP1	0.0159	0.0094	0.0180	3.9
WDR81	0.0238	0.0160	0.0414	0.6
XPO1	0.0280	0.0034	0.0086	2.7
ZCCHC10	0.0098	0.0039	0.0459	4.0

ZFAND2A	0.0355	0.0245	0.0142	0.5
ZFHX3	0.0008	0.0006	0.0157	0.2
ZNF124	0.0034	0.0022	0.0320	0.5
ZNF19	0.0017	0.0001	0.0460	0.1
ZNF264	0.0405	0.0244	0.0483	0.5
ZNF429	0.0048	0.0028	0.0451	0.2
ZNF438	0.0016	0.0014	0.0358	0.2
ZNF675	0.0177	0.0105	0.0497	0.5
ZSCAN18	0.0315	0.0081	0.0287	0.3
ZW10	0.0049	0.0043	0.0307	6.5
